# Meeting in the middle: motivational interviewing and self-determination theory

**DOI:** 10.1186/1479-5868-9-25

**Published:** 2012-03-02

**Authors:** William R Miller, Stephen Rollnick

**Affiliations:** 1 The University of New Mexico, Albuquerque, NM, USA; 2 School of Medicine, Cardiff University, Wales, UK

## 

Motivational interviewing (MI) is indeed a "bottom-up" model that emerged from practical experience in the field of alcohol treatment. The original description of MI [1] suggested some links to social psychological theories, but focused on an intuitive approach in treating alcohol problems for which there was at the time no empirical support. Our subsequent volumes [2,3] have similarly focused on clinical applications without proposing an underlying theory of treatment or change.

In part this reflects our own temperaments, preferring intuitive to rational-deductive ways of knowing [4,5], with a focus on the "real" world of clinical practice. We are "bottom-up" people. Much of what we have done in our careers has sprung from efforts to deal with practical problems that clinicians encounter in their daily work [6]. The world of academia, in contrast, tends to place a high premium on starting from coherent theory and rationally deriving hypotheses that will be tested to either confirm or revise the theory. This has simply never been a *forte *or primary scientific interest for either of us, to the dismay of some of our mentors and colleagues. We have preferred instead to move between the context of discovery and the context of justification [7] - deriving intuitive hypotheses from clinical experience, submitting them to the verification of scientific method, and then going back to the drawing board to try again. Over time, this approach may lead to the development of a higher-order theory as a byproduct [8]. The rigor of scientific method is equally important in both approaches. They differ in the source of hypotheses: intuitive experience versus rational deduction from a pre-existing theory. Both approaches have value and a long tradition in the history of science. Whether either one is in some sense superior to the other is a value judgment that we do not wish to make.

The history of MI, however, does suggest potential value in beginning from clinical intuition. A large evidence base comprising more than 200 randomized clinical trials has emerged, showing positive effects (albeit inconsistent) across many health problem areas. Well before this evidence base accumulated, however, MI disseminated readily and rapidly by word of mouth among clinicians, who are drawn to it not just from the clinical trials but because, for the lack of a better term, they seem to "recognize" it. It feels intuitively sound based on their own experience. This kind of practice-based evidence is also important, and needs to be compared, tested and refined with clinical trials. Hall [9] suggested a similar two-way street in psychotherapy research with cultural minorities. Evidence-based treatments are worth trying in populations where they have not yet been tested [10], and there is also a need for scientific study of the intuitive interventions that have arisen from an indigenous culture's own wisdom and experience.

So what about Self-Determination Theory (SDT) that grew up independently from MI, but bears a certain family resemblance? There may be a natural fit [11]. MI has lacked a well-developed theory to rationalize its efficacy. SDT has focused less on refining specific clinical procedures for putting it into practice. A marriage may be premature, but the flirtation is not. The three human hungers emphasized in SDT - autonomy, relatedness, and competence - are all directly addressed in MI. More than most psychotherapies, MI assumes, respects, and implicitly relies on volition to instigate self-regulation [12]. The emerging psycholinguistic "mechanisms" of MI [8,13,14] can be linked to the more general development of volition and self-regulation through language [15]. The relational component of MI also appears to be important [8], consistent with SDT. Supporting autonomy is a key element in the underlying spirit of MI. SDT and MI, it would seem, have much to learn from each other.

SDT also holds promise for improving our understanding of MI. A puzzling aspect in MI clinical research is the inconsistency of its outcomes. There are many positive trials, but also an impressive number of negative trials, including some of our own [16]. SDT may help to clarify the conditions that contribute to the effectiveness of MI in practice. MI has been faulted for underemphasizing social context [17], a factor that is clearly integrated in SDT, and a well-supported theory never hurts the academic credibility of any psychotherapy.

Is SDT more than just another pair of theoretical glasses through which to view the phenomena of MI? Will SDT lead to unique testable hypotheses that teach us important things about MI that we didn't already know? It remains to be seen. We do not propose to develop such a systematic integration ourselves, but we gladly offer our support to those whose aptitudes and inclinations lie in this direction.
